# Barriers to endocrine therapy adherence: perspectives of Black breast cancer survivors and their providers

**DOI:** 10.1007/s11764-024-01574-7

**Published:** 2024-03-23

**Authors:** Kimberley T. Lee, Brian D. Gonzalez, Carley Geiss, Hayden J. Fulton, Dannelle Charles, Susan T. Vadaparampil, N. Lynn Henry, Heather S. L. Jim, Dawn L. Hershman, Shelley S. Tworoger, Clement K. Gwede

**Affiliations:** 1 Moffitt Cancer Center and Research Institute, 10920 N Mckinley Dr, Tampa, FL 33612 USA; 2 University of Michigan, 1500 E. Medical Center Drive, Cancer Center, Room 7216, Ann Arbor, MI USA; 3 Herbert Irving Comprehensive Cancer Center, Columbia University Irving Medical Center, 161 Ft Washington Ave #1071, New York, NY USA

**Keywords:** Breast cancer, Endocrine therapy, Black, Disparities, Qualitative research

## Abstract

**Purpose:**

Adherence to oral endocrine therapy (ET) remains an issue for up to half of women prescribed these medications. There is emerging data that Black breast cancer survivors (BCS) have lower rates of ET adherence. Given the disparities in breast cancer recurrence and survival for Black BCS compared to their White counterparts, the goal of this study is to better understand barriers to ET adherence among Black BCS from the patient and provider perspectives.

**Methods:**

In this qualitative study, we conducted semi-structured interviews between October 29, 2021, and March 1, 2023. Interviews were recorded and transcribed, and coded data were organized into primary and secondary themes. Participants were recruited from a single academic cancer center. A convenience sample of 24 Black BCS and 9 medical oncology providers was included. Eligible BCS were 18 years or older, English-speaking, diagnosed with stage I–III hormone receptor-positive breast cancer, who had initiated ET.

**Results:**

Mean age of the BCS was 55 years (interquartile range, IQR 17 years). About one-fourth had a high school diploma or less (26.1%) and 47% completed a college education or higher. Approximately one-third of participants had annual household incomes of $40,000 or less (30.4%) or more than $100,000 (30.4%). Forty-three percent of the patient participants had private insurance; 11% were insured through Medicaid or the federal healthcare exchange; 26.1% had Medicare; and 13% were uninsured. Of the 9 medical oncology providers interviewed, 2 were advanced practice providers, and 7 were medical oncologists.

We found 3 major themes: (1) Black BCS often had concerns about ET before initiation; (2) after initiation, both BCS and providers reported side effects as the most impactful barrier to ET adherence; and (3) survivors experienced challenges with managing ET side effects.

**Conclusions:**

Our results suggest that multifaceted support interventions for managing ET-related symptoms may lead to improved adherence to ET among Black women and may reduce disparities in outcomes.

**Implications for Cancer Survivors:**

Multifaceted support interventions for managing ET-related symptoms may lead to improved adherence to ET among Black breast cancer survivors.

**Supplementary Information:**

The online version contains supplementary material available at 10.1007/s11764-024-01574-7.

## Introduction

Adherence to endocrine therapy (ET) for early-stage hormone receptor-positive (HR +) breast cancer (BC) saves lives. If taken daily for 5 years, ET reduces the risk of BC-related death by 50% [1–3]. However, nonadherence to ET remains an important clinical issue and worsens BC survival [4]. About half of BC survivors (BCS) underuse ET, via noninitiation, nonadherence, or early discontinuation [4, 5]. Furthermore, Black BCS are up to 50% less likely to adhere to ET compared to their White counterparts [6–10], which may worsen disparities in BC outcomes for Black BCS. Black women have an estimated 41 to 66% higher risk of BC recurrence or death than non-Black women [11, 12]. Black women are also more likely to develop metastatic BC with 9-year distant relapse-free survival rates of 89.1% for Black women vs 92.3% for White women [12]. These disparities in BC outcomes underscore the importance of increasing adherence to ET among Black BCS [13].

Despite the low rates of adherence to ET and resulting worse outcomes, limited interventions are available to improve adherence to ET. In addition, most interventions that have been tested have not demonstrated significant improvement in adherence to ET [14–18]. Of the published studies, none targets Black women. The goal of this study is to better understand barriers to ET adherence among Black BCS from the patient and provider perspectives.

## Methods

We conducted semi-structured interviews via videoconference with 24 Black BCS and 9 medical oncology providers from a single academic Cancer Center in southwest Florida which serves over 47% of Florida’s total population. The proportion of Black patients in our catchment area is lower than in the USA, the proportion of individuals living below the poverty level is similar to the US, while the uninsured in the catchment area is higher than in the US. Eligible BCS (1) were at least 18 years old, (2) were English-speaking, (3) were diagnosed with estrogen receptor and/or progesterone positive stage I–III invasive BC, (4) had completed all recommended definitive BC treatment (e.g., surgery, chemotherapy, radiation), (5) had initiated ET for BC, and (6) were able to provide informed consent. We sought to recruit BCS with diverse ages and experience with ET. Clinicians from the program helped identify potential participants. Eligible providers were healthcare professionals who provide clinical care to BCS. Recruitment was stopped when thematic saturation, the point when no new themes emerged for each group, was reached [19]. All participants were provided with a $50 gift card. This project was approved by Advarra’s Institutional Review Board.

### Data collection and analysis

Four study-team members with training and experience with qualitative research and with no clinical relationship to any participants (CG, HF, DC, BA) conducted interviews ranging from 40 to 70 min between October 29, 2021, and March 1, 2023. The interview guide for BCS asked questions about decision-making pertaining to initiating and discontinuing ET, why ET was being used in terms of risk–benefit profile, and challenges with taking ET (Supplementary materials). Provider interviews focused on understanding how providers counsel BCS about the need for ET, perceived barriers for BCS to remain adherent to ET, and recommendations for resources to help with ET adherence. All interviews were audio recorded, transcribed verbatim, and de-identified. During regular meetings, the study team discussed ongoing analysis, preliminary findings, and thematic saturation. NVivo 12 software [20] was used for analysis guided by tenants of applied thematic analysis [19]. Study-team members (CG and HF) developed a codebook with a priori codes and definitions refined through multiple rounds of iterative coding to achieve acceptable intercoder reliability (Cohen’s kappa = 0.89) [21]. Following line-by-line coding of all transcripts, coded data were organized into primary and secondary themes.

## Results

Twenty-four Black BCS participated in the study. Their median age was 55 years (IQR 17 years). About one-fourth had a high school diploma or less (26.1%); 26.1% had some college education; 17.4% completed a college education; and 30% had graduate degrees (Table 1). Approximately one-third of participants had annual household incomes of $40,000 or less (30.4%), $40,000 to $100,000 (34.7%), or more than $100,000 (30.4%). Forty-three percent of the patient participants had private insurance; 11% were insured through Medicaid or the federal healthcare exchange; 26.1% had Medicare; 13% were uninsured; and 4% had another insurance type. Of the 9 medical oncology providers interviewed, 2 were advanced practice providers, and 7 were medical oncologists.

**Table 1:** Demographic characteristics of Black breast cancer survivors

Characteristic	*N* (%)
Age in years, median (IQR)	55 (17)
Education	
High school diploma or less	6 (26.1)
Some college	6 (26.1)
College graduate or graduate degree	11 (37.7)
Annual household income	
Less than $40,000	7 (30.4)
$40,000–100,000	8 (34.8)
More than $100,000	8 (34.8)
Insurance type	
Private insurance	10 (43.5)
Medicaid/Federal Health Exchange	3 (13.0)
Medicare	6 (26.1)
Uninsured	3 (13.0)
Other	1 (4.3)

Qualitative analysis revealed 3 major themes: (1) Black BCS often had concerns about ET before initiation; (2) after initiation, both BCS and providers reported side effects as the most impactful barrier to ET adherence; and (3) survivors experienced challenges with managing ET side effects. These are described further within and illustrated using representative quotes.

### Theme 1: Black BCS often had concerns about ET before initiation

Participants described a range of concerns regarding beginning and continuing ET. While these concerns did not lead participants to decline or stop ET, for at least one participant, her concerns led her to delay initiating treatment: “I think I also delayed when I started taking my medicine. Like once I was cleared to start it back, I delayed probably another 3 weeks to start it. Just because … kind of dreading the side effects.”

#### Anticipating side effects

Side effects were reported as the major concern of participants when starting ET, including impact on quality of life (QOL), ability to work, and independence. For example, one patient said:

“You know, I was kinda skeptical because I was like well, I mean I want to survive but I want to enjoy my life. And it’s like do I take this? I mean, should I even try this medicine? Because I don’t want to be stuck in the bed. I don’t want to be sitting here and I can’t take care of my children; that if my grandchildren come over, I can’t spend time with them. I didn’t want to be put in a situation where I couldn’t take care of myself physically, even with just small daily activities or anything like that.”

#### Concerns about side effects compounded by treatment duration

ET is usually prescribed for a minimum of 5 years and can be extended up to 10 years. Participants were concerned not only with the long-term commitment of taking ET every day but also expressed uneasiness regarding possible long-term toxicities beyond expected side effects. One patient commented:

“When the doctor said that you gotta take this tamoxifen for 5 years, I was like, 5 years is too long to be doing any damn thing without it being either a habit or addictive or being permanently engrained in your DNA or whatever, right?”

#### Concerns about fertility

For participants who had not yet undergone menopause, ET’s impact on future fertility was part of the decision-making regarding the initiation of ET. One participant expressed this concern, although she indicated no plans to have children in the future: “We have 2 children, not saying that I want another child, but I just didn’t want that option to be completely stripped from me.” For another participant, ET drastically changed expectations for her future family in a negative way: “And they said that I would have to do an additional 5 years of hormone suppression. I think that was a little depressing to me because I had anticipated having more children.”

#### BCS want to put cancer in the “rearview mirror”

As described by providers, some BCS view daily ET as a reminder that cancer is still a risk in their life. One provider stated, “Taking the medication is a reminder to them about their breast cancer diagnosis. And sometimes they try to cope with that experience by trying to put it in the rearview mirror and try to kind of forget about it. And the treatment is a reminder of that. So, in some cases, they just want to put it behind them and ignore the potential consequences of that too.”

Another said, “Because I think everybody has an expectation that once they get through the big bulk of their treatment, they just wanna go back to the way they were before they were diagnosed with breast cancer … So, I think a good reason for the hard stop for all of my [racial/ethnic minority] patients that have been on ET is they cannot do this anymore. They would rather take the particular risk, no matter how small or large that might look like, in order to establish some normalcy—whatever that translates to them.” This theme was not borne out in BCS interviews.

### Theme 2: After initiation, both BCS and providers reported side effects as the most impactful barrier to ET adherence

Providers discussed how side effects are most troublesome for patients as side effects impact overall QOL. BCS provided examples of how side effects have negatively impacted their QOL, specifically related to relationships, sex life, overall outlook, body image, activity level (e.g., exercise, household chores), and work-life (Table 2). Side effects described as particularly impactful to patient QOL were joint pains, hot flashes, vaginal dryness, diminished sex drive, mood changes, brain fog, and sleep disturbances.

**Table 2:** Impact of endocrine therapy side effects on Black BCS

Domain of impact	Representative quotes
Daily activities	“I had some very hot flashes. It was very miserable… I had a lot of itching from it. Like, when you feel needles all over your skin sticking you. It was so bad that I could not even go out during the day. I would have to stay inside because as long as I had close contact to heat, the sun, the hot flashes along with the itching of my skin, it was awful. So, I would have to wear long clothes or not go outside during the day.” *“*I have insomnia, but I'll take something, like a ZzzQuil or Benadryl or something. But still, I'm just, if I'm not getting good sleep, I'll get up at like, 3 in the morning, and go back to sleep. I'm getting up a lot later. So, it's interfering with my normal daily routine.” *“*Also, like I feel like toward the end of the day I just feel, still feel kind of wiped out. So, like my energy is just not there. Which affects like getting things done around the house, everything … I see more of how it affects the people at home.”
Relationships	“I think it’s affected my relationship with my husband. [I’m] not really interested in having sex anymore.” *“*I just did the best I can, I could, and kind of stay by myself. I mean, it was a little hard to do because I do have a significant other. And he tolerated me. I'll put it that way. Because I knew I was kind of intolerable. I didn't like being with myself. So, I’m sure nobody else liked being with me, as well … It was a trying time, I’ll put it that way.”
Mood and mental state	“There’s the forgetfulness, there’s the stress, there’s depression, there’s the weight gain, all of that, and I went through all of it … I can say it’s like when I’m trying to write a paper, when I’m trying to focus on classes and so forth, okay, it was just simply not convenient for me to do that because I have to weigh out what’s important to me and how do I sustain myself.”
Body image	*“*And I was never a very tiny, small woman to begin with, but on this medication, it is extremely difficult to lose weight. Extremely. And that’s been a big challenge for me … But, you know, when you’re dealing with something as serious as cancer, your vanity is one of the first things you lose. Because after being totally bald, not having any hair on my head anywhere, and breast cancer surgery which cuts you up like a jack o’ lantern … I can only speak for women. For women in particular, gaining weight is a big deal. It’s a big, big, big, big deal. And I know that some women will not take the medicine if they feel that it’s going to make them gain weight.”
Work life	*“*But I definitely think brain fog is one of them. And in my field, I speak in front of people, believe it or not. So, just kind of forgetting what you're saying sometimes. You’re making this great argument to the judge and your words aren’t coming out right.”

#### Impact of age on ET side effects and QOL

Patient participants often reflected on their age in relation to the impact that side effects have had on their QOL. Older BCS commonly normalized symptoms (e.g., hot flashes, pain, weight gain, cognitive decline) as part of aging. For example, one participant shared, “I didn’t notice any change by taking it. I don’t know if weight gain is anything, but I’m always saying you get to a certain age, and your metabolism suddenly stops on you, and you can miss a meal and gain a pound or 2.” Another said, “And let me also just add that getting older came into play because okay, you’re dozing off a little bit. But I’m not 20 years old, so I didn’t ascribe it to the medication. I just ascribed it to the fact that well, maybe this is what happens when you get to be this age.”

One 37-year-old patient participant demonstrated how ET side effects can be particularly challenging for younger, premenopausal patients. ET side effects impacted her activity level, overall sense of self, psychological state, sex life, and relationship—describing how she felt as though she was living in the body of an “80-year-old woman.” With regards to vaginal atrophy and painful intercourse, this participant also stated, “But I didn’t realize I was going to be dealing with that, again as I said, at the age of 40 when I’m still in my prime. My husband is still in his prime … But now, I’m 40 and I’m not close to [my eighties].”

Providers described how, based on their observations, younger BCS have a harder time coping with side effects of ET, partly because they expected symptoms of menopause later in life. Providers cited side effects as particularly impactful for younger BCS’ sense of self and psychological well-being as well as intimate relationships. One provider said, “The younger patients, I think, have a harder time with the side effects … And I don’t blame them, you know, they have to be on this for 5 years, and they’re so young. They’re like, ‘Why do I have to feel like this for—you know, I’m young, I shouldn’t be feeling this way.’” Another provider said, “And the younger women—yeah, a difference would be in libido. Because it’s important, actually, in both groups. But especially the 70-year-old women … in general, will complain less about libido, where the younger ones, it is bothersome. It’s a problem.” Yet another stated, “I think that there might also be some stigmas in terms of relationships…They want to feel like they are feminine—they are a woman. You can’t really necessarily continue to have that maintained if you are on adjuvant ET and now you’ve gained weight, or you have low sexual libido, or you have vaginal dryness, you have hot flashes…And so, all these sort of issues, I think, create overall poor compliance … to the regimen in my younger African American, younger [racial/ethnic minority] patients.”

### Theme 3: BCS experience challenges managing ET side effects

#### Lack of side-effect reporting

Providers discussed concerns about BCS being uncomfortable with speaking about the challenges of side effects with their care team. They described how BCS may not want to *bother* their care team with contact outside of their appointments, as well as how for these BCS, sexual and mental health side effects are taboo.

Providers described how Black BCS *complain less* than White BCS. They described how this could reflect that Black BCS were just more reluctant than White BCS to communicate problems, despite both groups experiencing similar side effects. One provider described how Black BCS are less likely to discuss sexual side effects: “I do think that my White women, for example, they might be more open or more aware or bring up more front problems like vaginal dryness or low libido that I think that my Black women usually generally don’t talk about that.”

BCS also discussed how they hesitate to express issues with providers, describing how Black people are more likely to be “written off” by providers. One patient-participant relates this hesitancy to historical context (Table 3).

**Table 3:** Survivor and provider perspectives on barriers to reporting ET-related side effects

Survivor quotations	Provider quotations
“At first, I didn’t wanna discuss about the anxiety and the—this is my own hang-up. I didn’t want people to think I was going crazy … Because some of the anxiety and the mental things I was feeling.”	“I don’t think a lot of women report [sexual dysfunction] because they might just be shy about it.”
“So, I think it’s just growing up in that time where the medical field wasn’t just that open to you. And you get stuck in that. Even though the times have changed, we can still get stuck in the past—in the way it was.”	“It’s hard to generalize, but I would say if anything, my African-American patients probably complain less about the side effects than the Caucasians.”

#### Lack of resources to manage ET side effects

Providers described being limited in how they can support BCS in managing ET side effects, primarily because of a lack of treatment options or available resources. Insomnia, dizziness, weight gain, sexual dysfunction, and brain fog were side effects mentioned as particularly challenging for providers to manage. Providers described how additional resources such as expanded mental health services, sleep specialists, and neurocognitive function specialists would benefit supporting adherence (Table 4).

**Table 4:** Provider perspectives on the need for resources for side effect management

“There is a very large population of women with anxiety and depression that is not diagnosed. We have some women who are debilitated by their mood disorders who cannot tolerate this treatment. And it would be really great if our psychosocial care team was large enough and equipped to help support them through their therapy, because we could probably prevent a lot of metastatic recurrence if we had that type of support.”
“I’ve seen patients on the brink of divorce. I’ve seen patients on the brink of losing their jobs. I mean, it’s been a lot. And then, I just sometimes feel like we could probably do a little bit more in that arena when it comes to adverse effects. It is the behavioral-emotional component that I think we might lack a bit in, and we could do better.”
“I don’t know to what extent there is support for sexual dysfunction. A lot of women don’t report that to me, but I know it’s a major issue. So, I think some of the resources are there, but I think some things could maybe be a little bit better.”

## Discussion

Our findings demonstrate that side effects from ET are a significant barrier to ET adherence among Black women. Side effects negatively impact several important domains of QOL for Black BCS (e.g., physical functioning, sexual functioning, mood). Our finding of side effects being a predominant barrier to ET adherence also replicates the findings of Spencer et al., which showed that Black BCS were more likely to skip ET pills because of side effects and concerns about long-term medication use [22]. Side effects as barriers begin at treatment initiation, as demonstrated by our findings that Black BCS have concerns about side effects before treatment initiation. Shared decision-making may represent one tool for providers to help patients address these and other concerns before starting ET. In a prospective cohort consisting of 553 Black BCS, Spencer and colleagues found that women who did not describe the decision to start ET as a shared decision with their provider were less likely to adhere to ET [22].

Given that ET is the mainstay of treatment for HR + BC, optimizing delivery of ET to Black women plays an important role in potentially mitigating disparities in outcomes among Black BCS. There are few prior studies that have investigated barriers to ET adherence among Black BCS. Patient-physician communication plays an important role in patient decision-making and side effect reporting and management. However, a 2017 systematic review of 17 studies found that Black patients experience a lower quality of communication with their physicians [23]. In oncology patient-provider interactions, oncologists are less likely to discuss side effects with Black patients, and Black patients are less likely to understand the information provided by their oncologist compared to non-Black patients [24]. We found that Black BCS may be less likely to report side effects related to sexual function. Wheeler and colleagues found similar findings, with Black women being less likely to report sexual side effects compared to White women [22]. Whether Black BCS have a higher risk of ET-related sexual side effects, or they experience more barriers to reporting these issues because of concerns about reporting them to their providers remains unclear.

The recommended duration of ET (5–10 years) can lead to significant out-of-pocket costs for medication and management of ET-related side effects. Racial disparities in BC care delivery are profound and occur across the cancer care continuum [25]. Black patients with BC are less likely to receive timely surgery, chemotherapy, and ET and are also more likely to receive nonstandard chemotherapy regimens and lower than recommended doses of chemotherapy [26–30]. Many of these treatment disparities contribute to disparities in outcomes for Black BCS [25–27, 31]. Support for managing ET side effects is difficult to access and incurs additional costs to patients. This support is not built into routine oncologic models of care and may further exacerbate disparities in care.

**Fig. 1:**
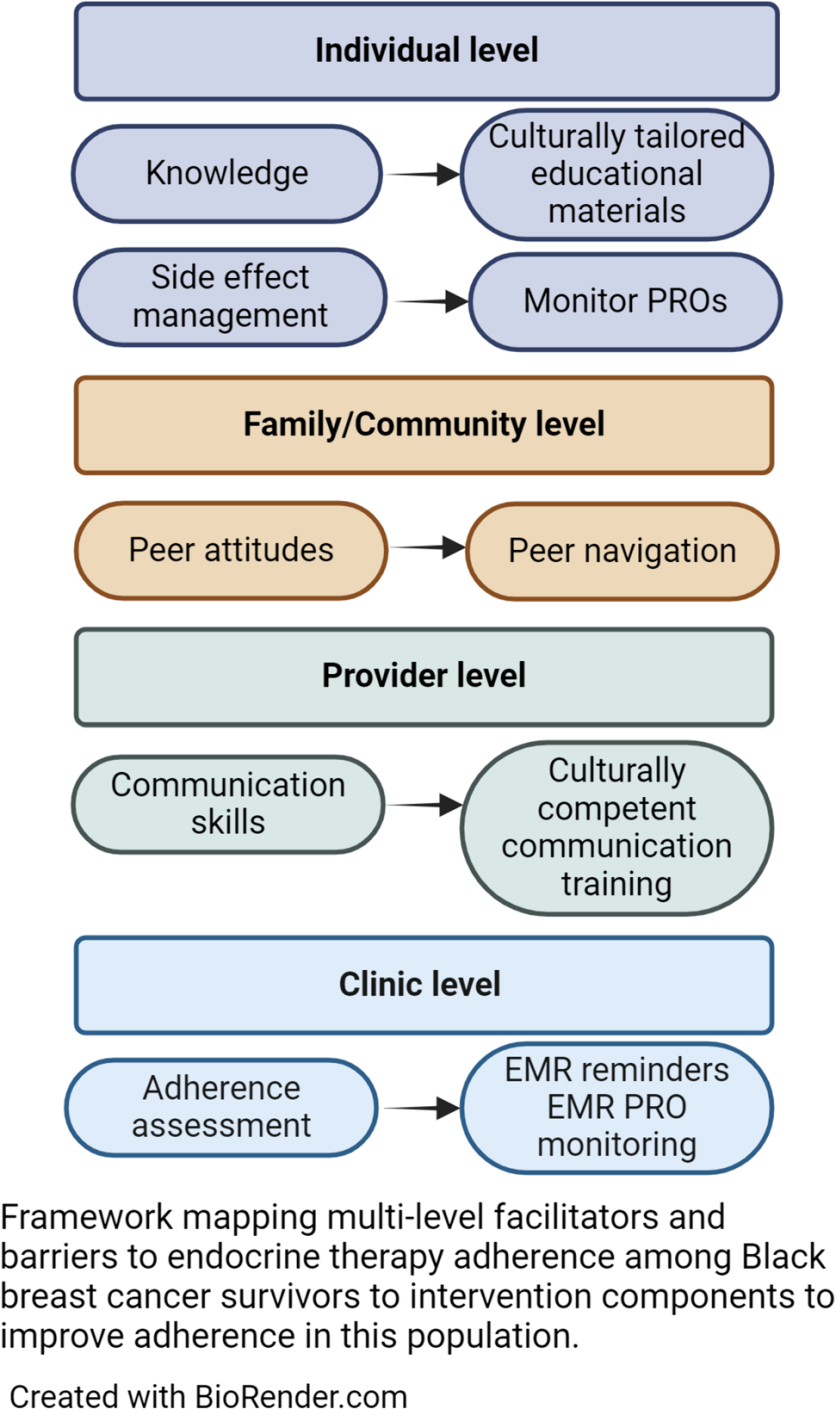
Framework mapping multi-level facilitators and barriers to endocrine therapy adherence among Black breast cancer survivors to intervention components to improve adherence in this population. Created with BioRender.com

Our results suggest that multilevel support interventions for managing ET-related symptoms may lead to improved adherence to ET (Fig. 1). Such interventions should include patient education about the importance of ET and symptom monitoring and management. At the community level, peer navigation or support groups may help to inform attitudes toward ET adherence. Provider training in culturally competent communication skills along with adequate resources for symptom management at the healthcare system level (e.g., integration of adherence assessments and patient-reported outcome tracking in the electronic medical record) would help to address system-level barriers to ET adherence among Black women. It is important that such interventions incorporate racial and cultural differences to address known disparities to BC care and outcomes.

### Limitations

The sample is drawn from Black BCS and providers from a single academic Cancer Center in Florida. This limits generalizability to women in other regions and who are served by different healthcare systems. Purposeful sampling used to improve thematic variation and small sample size may also limit the generalizability of these findings. This study only included BCS who initiated ET, which restricts the investigation of reasons for noninitiation. Participants received renumeration for their participation which may have introduced a selection bias favoring those with financial need. However, this work fills an important gap in the literature—focusing on a population with worse BC survival outcomes.

## Conclusions

Side-effects of ET are an important barrier to adherence among Black BCS. While this finding has been demonstrated in non-Black BCS populations [32], additional barriers to care faced by Black BCS require attention in interventions to address ET adherence disparities, such as cultural tailoring to ensure suitability of study materials for Black BCS.

## Supplementary Information

Below is the link to the electronic supplementary material.

Supplementary file1 (DOCX 17 KB)

Supplementary file2 (DOCX 25 KB)

## Data Availability

The datasets generated and analyzed during the current study are available from the corresponding author on reasonable request.
