# Supplementary material for: Barriers to endocrine therapy adherence: perspectives of Black breast cancer survivors and their providers
Source: J Cancer Surviv. 2024 Mar 23;19(5):1580–7. doi: 10.1007/s11764-024-01574-7 (PMC11664442; doi:10.1007/s11764-024-01574-7)
Supplement: Supplementary file 1 — Supplementary file1 (DOCX 17 KB) [file 11764_2024_1574_MOESM1_ESM.docx]

**Interview Guide for Clinic Personnel**

We are interested to learn about your experiences and perspective in prescribing adjuvant endocrine therapy (AET). We’re particularly interested in understanding the specific challenges Black women with non-metastatic, invasive breast cancer face. There is no right or wrong answer. Please do not feel like you have to answer in a certain way.

Do you have any questions about what I have told you so far? [answer questions]

Is it OK with you if I begin recording, so I’m sure to capture all of your input? [begin recording]

1. Can you please describe your experience in providing care to patients who receive AET?
2. What are some of the challenges patients have in general with taking AET? Are there any groups of patients who seem to have more challenges with taking AET?
3. Patients are diverse in terms of age, culture, race, comorbidities, medical distrust, and so forth. *How do these patient differences inform the way you tailor discussions regarding AET?*
4. Do you specifically counsel patients on risk of recurrence with or without treatment with endocrine therapy? *If so, how do you present this information to them?*

As I mentioned earlier, we’re particularly interested in learning more about the experiences of Black patients with AET. This next set of questions will ask about challenges and barriers Black women face in receiving AET.

1. For Black patients who decline or are hesitant to start endocrine therapy, what are the most common reasons? *Anything else?*
   1. Have you noticed any differences in reasons compared with White patients who decline or are hesitant to start AET?
2. What are some common barriers for Black women in taking their endocrine therapy everyday? *For staying on endocrine therapy for 5-10 years?*
3. What are some of the reasons Black patients decide to stop AET?
   1. Have you noticed any differences in reasons to stop AET compared with White patients?

Clinic Processes and Resources

1. How do you assess adherence (Whether or not patients take their endocrine therapy every day) in your practice?
2. What current resources at Moffitt help you assist your patients to take their endocrine therapy every day? *And stay on their endocrine therapy for 5-10 years?*
3. What resources would you like to have to help support your patients with taking their endocrine therapy every day? *And staying on their endocrine therapy for the full time it’s prescribed?*
4. Do you feel that you have all the resources you need to manage the symptoms your patients on endocrine therapy may develop? *What additional resources would you like to have access to?*
5. We’re considering how we could develop a clinic wide symptom monitoring program to help improve adherence to endocrine therapy. This may take the form of a mobile app that collects electronic patient related outcomes related to side effects of endocrine therapy or through patient navigators assessing symptoms between visits. What are your initial thoughts on something like this? *What might be some limitations to doing so? Are there any benefits to doing so?*

PREDICT is a free online tool that helps show how breast cancer treatments after surgery might improve survival rates. It’s like Adjuvant! Online. PREDICT has been endorsed by the American Joint Committee on Cancer. After inputting tumor and patient demographic characteristics, PREDICT gives data on absolute benefit of treatment being considered and overall survival. It provides this information in 5 different formats for the user to choose from – table, curves, chart, text, and icons. [Show PREDICT slides/Provider aids here]

1. How would you feel about incorporating decision aids such as *PREDICT* in your discussions with Black women regarding endocrine therapy? *Do you have any recommendations for how this should be done?*

Is there anything else I haven’t asked you that you would like to share to help me better understand issues associated with AET, for Black patients in particular or patients in general?
