# Supplementary material for: Barriers to endocrine therapy adherence: perspectives of Black breast cancer survivors and their providers
Source: J Cancer Surviv. 2024 Mar 23;19(5):1580–7. doi: 10.1007/s11764-024-01574-7 (PMC11664442; doi:10.1007/s11764-024-01574-7)
Supplement: Supplementary file 2 — Supplementary file2 (DOCX 25 KB) [file 11764_2024_1574_MOESM2_ESM.docx]

**Patient Interview Guide**

The study team is trying to improve the experience and outcomes of women, particularly Black women, on endocrine or hormonal therapy. These are pills such as tamoxifen, anastrozole/Arimidex, exemestane/Aromasin or letrozole/femara for treatment of breast cancer. We are interested to know your opinions about taking hormonal or endocrine therapy. More specifically, we hope to learn about your decision-making process, overall experience, and perspective. There is no right or wrong answer. Please do not feel like you have to answer in a certain way.

The last thing I want to mention is that I am not a medical doctor. As I said before, I’m not going to share anything you tell me with your treatment team. So, if you are having any health concerns, make sure to tell your treatment team directly.

Do you have any questions about what I have told you so far? [answer questions]

Is it OK with you if I begin recording, so I’m sure to capture all of your input? [begin recording]

1. To start off, can you briefly tell me about when you were first diagnosed with breast cancer?

PROBE: How has cancer impacted your life? What’s your experience been like working with your care team during your cancer treatment?

- 1. How would you describe the communication with your care team?
  2. How would you describe your level of trust toward your doctors and care team?
  3. Can you describe how comfortable or uncomfortable you felt interacting and communicating with your care team?

PROBE: What did they do/didn’t do to make you feel that way?

- 1. Can you tell me about a time when you had a negative experience with your care team?
  2. Do you feel there is anything that would have improved the dynamics with your care team?
  3. How did interactions with your cancer care team differ from previous experiences with healthcare providers?

1. How, if at all, do you think race and ethnic backgrounds influence how patients are treated in healthcare?
   1. Have you ever felt that your race or ethnic background influenced the dynamics between you and your cancer care team?
   2. Have you ever felt that your race or ethnic background influenced dynamics between you and *other* healthcare providers, outside of your cancer care?
   3. Do you feel like having a doctor of the same race or ethnicity would make you feel more comfortable throughout your cancer care? How so?

PROBE: What benefits do you think come from having a doctor of your same race and ethnicity?

- 1. Do you feel like having a woman doctor would make you feel more comfortable throughout your breast cancer care? How so?

1. How did you feel about the recommendation to begin hormone therapy?

PROBE: What was concerning or appealing about taking hormone therapy?

- 1. Was any aspect of hormone therapy left unclear or confusing?

1. What informational resources (from clinics, online, personal networks) about hormone therapy have you used to better help you understand the treatment?
2. What sorts of information or support did you get from [resource]? (e.g., information about side effects, social support)
3. Was there anything missing from your care that led you to look elsewhere (e.g., online)?
4. What have been the most helpful resources for you?
5. How did these resources inform your decisions about hormone therapy?
6. Are there any resources or information that you try to avoid? Why?
7. What has your overall experience been like taking hormone therapy?

PROBE: How long did you take it/have been taking it?

What’s been the most challenging part of taking hormone therapy?

1. What side effects if any did you have/are you having from therapy?
   1. How difficult were these side effects to manage or cope with?
   2. How did/do these side effects impact your life (e.g., work, relationships, family duties, household duties, sleep)?
   3. Did you ever stop therapy because of these side effects?

If no-> Did you ever *consider* taking time off therapy because of this?

1. What helped you get though these challenges with side effects? (e.g., family, faith)
   1. Did you ever discuss these challenges with your medical team? Why or why not?

PROBE: What did they recommend? Was it helpful?

- 1. Can you think of any topics or side effects you, or other patients, wouldn’t feel comfortable discussing with your medical team?

1. It’s very common for patients to stop taking pills for a period of time. Can you tell me about a time this happened to you?

PROBE: What caused you to stop taking the pills?

PROBE: Did symptoms or side-effects ever cause you to stop taking the pills?

- 1. Was there a time you considering stopping but didn’t?

**Only for those who stopped hormonal therapy prior to 5 years:**

1. Can you tell me how you made the decision to stop taking hormonal therapy?
   1. What are some of the reasons why you made this decision? Anything else?

PROBE: Did you have any symptoms that influenced your decision to stop hormonal therapy?

- 1. What information helped with this decision? (e.g., information provided by doctors, family, support group)

1. Can you describe the conversation with your doctor or care team about ending hormone therapy?

PROBES: How was this idea brought up? What was their response?

- 1. Can you describe if your doctor or other care team member offered any suggestions to help with your concerns?
  2. Is there anything you wish could have been different about this decision-making?

1. What’s your perspective on the risk of the cancer coming back, since you’ve stopped hormone therapy?

**For everyone- improvements and interventions:**

*We are interested in coming up with ways to help patients, particularly Black women, improve their experience and stay on the hormonal therapy pills as prescribed- and we’d like to get your opinion on how we can best support patients to do so.*

1. What is something that your care team could have done differently to better support you during hormone therapy?
   1. Hormone therapy can be really confusing to understand. Can you think of ways providers can more clearly educate patients on how it works?

PROBE: What has been most confusing about hormone therapy? How it works? The benefits? The risks?

1. What advice would you give medical providers prescribing this medication, so they can better support and improve care for Black Women?
2. Are there any additional services you think should be available (e.g., through Moffitt) in order to help women on these medications? Eg. A structured way to monitor your symptoms, easier referrals to help with symptom management, reminders to take the medication
3. In addition to helping with symptoms, what other issues do you think need to be addressed to help women take these medications as prescribed?
   1. Do you see any specific issues facing Black Women?

PROBE: These might be issues that you have faced, those people you know have faced, or community issues that could influence the experience on hormone therapy.

1. Is there anything else I haven’t asked you about this topic that you would like to share to help me better understand your experience?
